# Country data on AMR in Vietnam in the context of community-acquired respiratory tract infections: links between antibiotic susceptibility, local and international antibiotic prescribing guidelines, access to medicines and clinical outcome

**DOI:** 10.1093/jac/dkac214

**Published:** 2022-09-06

**Authors:** Didem Torumkuney, Subhashri Kundu, Giap Van Vu, Hoang Anh Nguyen, Hung Van Pham, Praveen Kamble, Ngoc Truong Ha Lan, Nergis Keles

**Affiliations:** GlaxoSmithKline, 980 Great West Road, Brentford, Middlesex TW8 9GS, UK; GlaxoSmithKline, 23 Rochester Park, 139234Singapore; Respiratory Center, Bach Mai Hospital, Hanoi 100000, Vietnam; The National Centre for Drug Information and Adverse Drug Reactions Monitoring, Hanoi University of Pharmacy, Hanoi, Vietnam; University of Medicine and Pharmacy, 217 Hong Bang Street, Ward 11, District 5, Ho Chi Minh City, Vietnam; GlaxoSmithKline, 252, Dr Annie Besant Road, Worli, 400030 Mumbai, India; GlaxoSmithKline Vietnam, Unit 701, 235 Dong Khoi, District 1, Ho Chi Minh City, Vietnam; GlaxoSmithKline, Büyükdere Cad. No: 173, 1. Levent Plaza B Blok 34394 Levent, Istanbul, Türkiye

## Abstract

**Background:**

Antimicrobial resistance (AMR) is one of the biggest threats to global public health. Selection of resistant bacteria is driven by inappropriate use of antibiotics, amongst other factors. COVID-19 may have exacerbated AMR due to unnecessary antibiotic prescribing. Country-level knowledge is needed to understand options for action.

**Objectives:**

To review the current situation with respect to AMR in Vietnam and initiatives addressing it. Identifying areas where more information is required will provide a call to action to minimize any further rises in AMR within Vietnam and improve patient outcomes.

**Methods:**

National initiatives to address AMR in Vietnam, antibiotic use and prescribing, and availability of susceptibility data, in particular for the key community-acquired respiratory tract infection (CA-RTI) pathogens *Streptococcus pneumoniae* and *Haemophilus influenzae*, were identified. National and international antibiotic prescribing guidelines for CA-RTIs (community-acquired pneumonia, acute otitis media and acute bacterial rhinosinusitis) commonly used locally were also reviewed, plus local antibiotic availability. Insights from clinicians in Vietnam were sought to contextualize this information.

**Conclusions:**

In Vietnam there have been some initiatives addressing AMR; Vietnam was the first country in the Western Pacific Region to develop a national action plan to combat AMR, which according to the WHO is being implemented. Vietnam also has one of the highest rates of AMR in Asia due, in part, to the overuse of antimicrobial drugs, both in the animal health sector and in humans in both hospitals and the community. In addition, despite a 2005 law requiring antibiotic prescription, there is unrestricted access to over-the-counter antibiotics. Several global surveillance studies provide antibiotic susceptibility data for CA-RTI pathogens in Vietnam including Survey of Antibiotic Resistance (SOAR) and SENTRY (small isolate numbers only). For management of the common CA-RTIs in Vietnam there are several country-specific local antibiotic prescribing guidelines and in addition, there is a range of international guidelines referred to, but these may have been created based on pathogen resistance patterns that might be very different to those in Vietnam. Expert clinician opinion confirms the high resistance rates among common respiratory pathogens. A more standardized inclusive approach in developing local guidelines, using up-to-date surveillance data of isolates from community-acquired infections in Vietnam, could make management guideline use more locally relevant for clinicians. This would pave the way for a higher level of appropriate antibiotic prescribing and improved adherence. This would, in turn, potentially limit AMR development and improve clinical outcomes for patients.

## Introduction

Antimicrobial resistance (AMR) is one of the biggest threats to public health throughout the world^1^ as described in the introductory paper of this Supplement.^2^ The WHO states that ‘the world urgently needs to change the way it prescribes and uses antibiotics. Even if new medicines are developed, without behaviour change, antibiotic resistance will remain a major threat’.^3^ The first paper in this Supplement included details about the multiple factors which can drive a rise in AMR along with the global initiatives that are in place to address this threat.^2^ Each country and/or region must also play their part through local initiatives.

In order to identify how AMR can be addressed in Vietnam in the future, it is necessary to review what is happening now. In this paper, we present the current situation in Vietnam, determined by using published information (from searching PubMed, Google Scholar and other internet platforms) to ascertain any national initiatives to address AMR, antibiotic use and prescribing, and availability of susceptibility data, in particular for the key community-acquired respiratory tract infection (CA-RTI) pathogens *Streptococcus pneumoniae* and *Haemophilus influenzae*. National and international antibiotic prescribing guidelines for CA-RTIs, specifically community-acquired pneumonia (CAP), acute otitis media (AOM) and acute bacterial rhinosinusitis (ABRS), commonly used by healthcare professionals in Vietnam were also reviewed, along with how these link to local antibiotic availability. Insights from a clinician in Vietnam were sought to contextualize this information. In addition, we aimed to identify areas where more information is required and present a call to action to improve clinical outcome for patients and to minimize further rises in AMR within Vietnam.

## Action Plans

Following the formulation by the World Health Assembly in 2015 of a Global Action Plan (GAP) for AMR^4^ many countries began to develop their own National Action Plan (NAP). For the future, enhanced regulation, patient/public education, provision of information on local resistance patterns, establishing antimicrobial stewardship programmes and developing national and regional consensus programmes have been proposed to help combat AMR.^5^ Vietnam was the first country in the WHO Western Pacific Region to develop a NAP to combat AMR. It was produced by the Ministry of Health in 2013 and entitled the ‘National Action Plan on Combating Drug Resistance in the Period 2013–2020’.^6,7^ The plan focused principally on human health and a further plan was developed to combat drug resistance in livestock and aquaculture. Antibiotic use in food production is perceived as a key driver of AMR in humans. As addressing AMR requires a coordinated approach, relating to not only human use, but also to the use of antibiotics in animals and agriculture, there is potential to strengthen multi-sectorial collaboration between the animal and human health sectors and create cross-disciplinary awareness and participation, to provide a One Health Approach to strengthen the animal health sector and reduce the spread of AMR.^8^ According to the report by the WHO for 2020–21, Vietnam’s NAP is being implemented.^9^

## Antibiotic usage

Vietnam is currently classified as a lower middle-income country with a population of 94.6 million and GDP per capita of US $2171. In 2016, health expenditure accounted for 5.7% of GDP. Antimicrobials accounted for one-third of the total spend on medications in public hospitals in Vietnam and currently there are many different forms and formulations of antimicrobials available on the Vietnamese market.^10^

Vietnam has one of the highest rates of AMR in Asia. In an AMR surveillance study using a network of 16 hospitals in Vietnam between 2012 and 2013, the proportion of AMR was high among all pathogens isolated from clinical specimens and in most, resistance had increased since an analysis in 2009.^11^ That study emphasized the need for more surveillance of antibiotic resistance.^11^ In rural Vietnam, a study of resistance of *S. pneumoniae* in preschool children reported that multidrug resistance increased from 31% to 80% over a 15 year period (1999–2014).^12^

Several factors have contributed to the increase in AMR. In Vietnam, there is substantial overuse of antimicrobial drugs, both in the animal health sector and in humans in both hospitals and the community and this is an important driver for the emergence and spread of AMR.^11^ A study of antibacterial consumption in 76 countries between 2000 and 2015 found that Vietnam ranked 11th in antibacterial consumption with 32.0 DDD per 1000 inhabitants per day; this is a higher rate of prescribing than that seen in most EU countries.^13^ Another study extracted antimicrobial usage data for key animal species and humans from different published sources. Total antimicrobial use in Vietnam equated to 261.7 mg/kg and 247.3 mg/kg of human and animal biomass, respectively, compared with 122.0 mg/kg and 151.5 mg/kg in the EU.^14^ A review of the consumption of antibiotics in rural Vietnam, in relation to the WHO Access, Watch, Reserve classification showed a high consumption of antibiotics from the Watch group, particularly in children. Watch group antibiotics were provided frequently from private pharmacies showing the requirement for putting into practice pharmacy-specific interventions.^15^

Despite a 2005 law requiring antibiotic prescription, there is unrestricted access to over-the-counter (OTC) antibiotics in Vietnam, with 38% of caregivers still accessing antibiotics without any formal medical assessment. Limited knowledge amongst members of the public is also an issue, with many people buying antibiotics to treat the common cold.^5^ Consumers of antibiotics in Vietnam are influenced by what constitutes a high-quality medicine and give less emphasis to the importance of a correct diagnosis. They regard antibiotics as a ‘trusted remedy’ but have a limited understanding of antibiotic resistance. Social media also influences consumption and choice of antibiotics.^16^

A large proportion of inpatients are reported as receiving inappropriate antibiotic treatment in Vietnamese hospitals.^5^ Unnecessary hospitalization has been reported in children with non-severe pneumonia and there is a reluctance to step down from intravenous to oral antibiotics.^17^

Several physician-related factors also contribute to the overuse of antibiotics, including patient pressure, lack of access to microbiology services and limited knowledge of local resistance patterns.^5^

## Surveillance

Pneumonia is the most common infection in the community and *S. pneumoniae* and *H. influenzae* are the two most common causative pathogens, both in children and adults. This has been confirmed by various investigations in Vietnam including a recent study which made use of combined culture and real-time PCR results to investigate the pathogens causing CAP in Vietnamese outpatients. Microbial pathogens were detected in 94% of cases, of which 18.7% involved single pathogens, including *S. pneumoniae* (5.2% of all cases) and *H. influenzae* (7.8% of all cases). Multiple pathogens were detected in 76.3% of cases and most of these were *S. pneumoniae* and *H. influenzae* and/or combined with other pathogens; *S. pneumoniae* and other pathogens; and *H. influenzae* and other pathogens.^18^ For this reason antibiotic susceptibility of these two pathogens is the focus of surveillance studies in the community.

### Global surveillance studies

#### SOAR

Several global surveillance studies provide antibiotic susceptibility data in Vietnam. The Survey of Antibiotic Resistance (SOAR) is a multinational antibiotic surveillance study, ongoing in an expanding range of countries since 2002. The study aims to collect and make available in published, peer-reviewed papers, antibiotic susceptibility data, specifically for *S. pneumoniae* and *H. influenzae,* the most commonly isolated respiratory bacterial pathogens in the community.^19^ Key features of SOAR are that it focuses on only these pathogens, and identification and susceptibility testing are performed in an independent centralized laboratory using standardized methodology (CLSI) allowing for comparisons to be made between countries/regions and for the identification of trends over time. SOAR data is analysed based on three different breakpoints: CLSI, EUCAST dose-specific, and PK/PD breakpoints.

Clinical breakpoints are cut-off MIC values used to classify microorganisms into the clinical categories susceptible (S), intermediate (I) and resistant (R) to assist in the prediction of the clinical success or failure of a specific antibiotic. Two international organizations define breakpoint values: CLSI and EUCAST. Due to variation in criteria for their definition, there are some differences between CLSI and EUCAST in the clinical breakpoint values for certain bacteria for some antibiotics and this can impact susceptibility interpretation of clinical isolates.^20,21^ EUCAST breakpoints are dose-specific and use the EMA-approved doses that are included in the Summary of Product Characteristics of an antibiotic. This means that by application of breakpoints for higher doses, the effect of using a raised dose on the clinical efficacy of a particular antibiotic can be predicted. Currently, most clinical microbiology laboratories in Vietnam use CLSI breakpoints, however the international application of the EUCAST breakpoints is expanding,^22^ so it is possible that dose-specific breakpoints could at some time also be applied in Vietnam. The EUCAST dose-specific breakpoints can also be used retrospectively to calculate the susceptibility of previously collected isolates to show the susceptibility levels that would have been achieved at higher doses.

Use of the EUCAST dose-specific breakpoints shows the effect of increasing the antibiotic dose on the susceptibility of a pathogen, providing additional information so the prescriber can decide if a higher dose would be of benefit. For example, *S. pneumoniae*, the most commonly isolated respiratory bacterial pathogen^23,24^ for infections such as CAP, AOM and ABRS has over time become less susceptible to amoxicillin/clavulanic acid in some countries^25^ since the MIC of some isolates has increased. When treating infections, it is important to be able to eradicate bacterial pathogens with raised MICs to optimize the clinical outcome while at the same time minimizing the risk of selecting variants with even higher MICs. This is possible because β-lactams, unlike many other antibiotics, have time-dependent killing properties. Their efficacy depends on the amount of time an adequate drug concentration is present at the site of action. Although increasing the concentration at the infection site above a particular concentration will not have any effect on the efficacy, the use of higher doses and/or more frequent dosing allows for successful eradication of infections caused by pathogens with higher MICs because the time the antibiotic is above the MIC is increased.^26^

**Figure 1.**
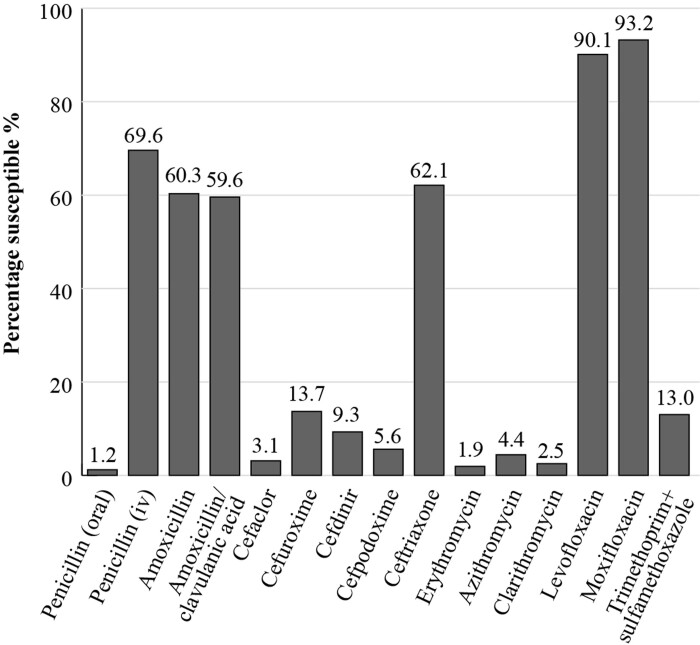
Percentage susceptibility rates for *S. pneumoniae* isolates (*n = *161) collected as part of the SOAR study in Vietnam in 2016–18 based on CLSI breakpoints.

The most recent SOAR results for Vietnam, from pathogens isolated from samples collected at two sites in 2016 to 2018 from outpatients with confirmed CA-RTIs, show that when applying the CLSI breakpoints, 59.6% of *S. pneumoniae* isolates (*n*  = 161) were susceptible to amoxicillin/clavulanic acid but susceptibility fell to 4.4%, 2.5% and 1.9%, respectively, for the macrolides azithromycin, clarithromycin and erythromycin. Susceptibility to cephalosporins (cefaclor, cefdinir, cefpodoxime, and cefuroxime) was also generally low, except for ceftriaxone for which susceptibility was 62.1%. For the fluoroquinolones levofloxacin and moxifloxacin, *S. pneumoniae* susceptibility was 90.1% and 93.2%, respectively (Figure 1). Applying PK/PD breakpoints, *S. pneumoniae* susceptibility to amoxicillin (1.5 g/day) was 60.3%, to cefixime and cefpodoxime, both 5.6%, to ceftriaxone, 62.1% and to azithromycin, 2.5%.^19^

**Figure 2.**
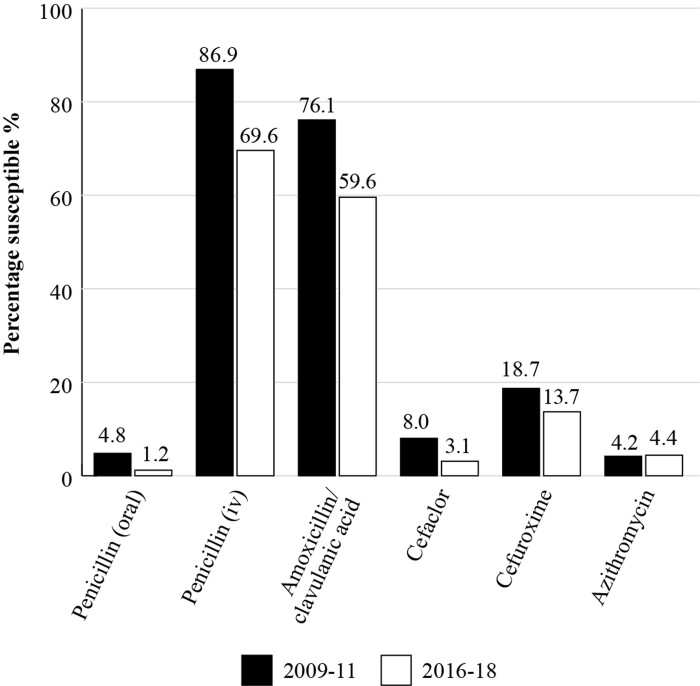
Percentage susceptibility rates for *S. pneumoniae* isolates corrected as part of the SOAR study in Vietnam in 2009–11 (*n  = *289) and 2016–18 (*n  = *161) based on CLSI breakpoints.

By using SOAR data from different time periods, susceptibility trends over time can be identified. Figure 2 is a comparison of susceptibility of *S. pneumoniae* isolates in Vietnam for 2009–11 with the results for isolates collected in the period 2016–18 and shows that *S. pneumoniae* isolates had become less susceptible to penicillin, amoxicillin/clavulanic acid, cefaclor and cefuroxime during that time period.^19,27^*S. pneumoniae* susceptibility to azithromycin was consistently low across both time periods.^19,27^

**Figure 3.**
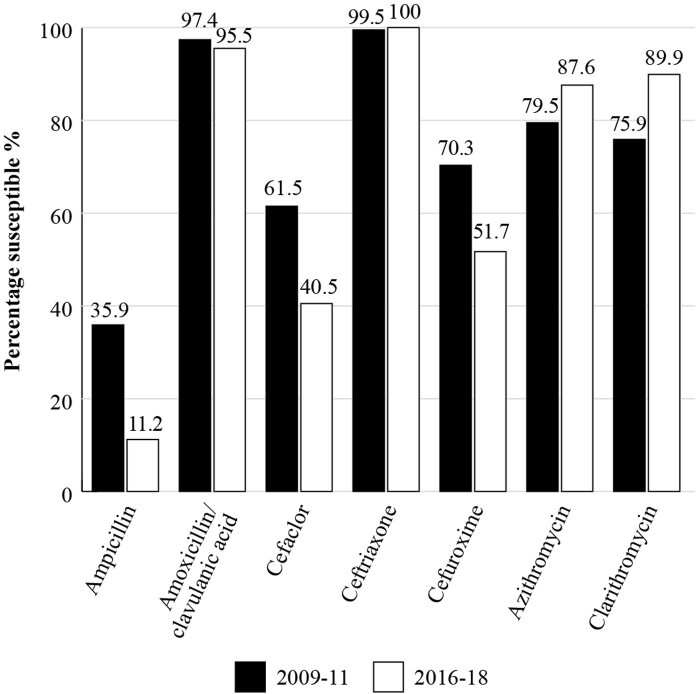
Percentage susceptibility rates for *H. influenzae* isolates collected as part of the SOAR study in Vietnam in 2009–11 (*n  = *195) and 2016–18 (*n  = *89) based on CLSI breakpoints.

For all *H. influenzae* isolates (*n  *= 195, 2009–11; *n  *= 89, 2016–18) from Vietnam, a high susceptibility of 97.4% to amoxicillin/clavulanic acid was seen in 2009–11, using CLSI criteria (Figure 3) and this level of susceptibility was maintained in 2016–18 at 95.5%. Figure 3 shows the susceptibility trends for *H. influenzae* by comparing the two time periods. Susceptibility to ampicillin was 35.9% by CLSI criteria in the early period and fell to 11.2% in 2016–18, reflecting the prevalence of β-lactamase-positive isolates. There was also a fall in susceptibility to cefaclor and cefuroxime over the latter time period.^19,27^ In the 2016–18 time period, the susceptibility of *H. influenzae* isolates (using CLSI criteria) to cefixime and cefpodoxime was 70.8% and 60.4%, respectively.^19^

#### SENTRY

The SENTRY Antimicrobial Surveillance Program was initiated in January 1997 and was designed to monitor the predominant pathogens and AMR for nosocomial and community-acquired infections globally. Data are available for susceptibility of *S. pneumoniae* isolates collected in Vietnam in 2018 but the number of isolates was very low (*n  *= 9).^28^

#### GLASS

In 2015, the WHO launched the Global Antimicrobial Resistance and Use Surveillance System (GLASS). GLASS is the first global system to collect national AMR data for selected bacterial pathogens that cause common infections. The aim is to monitor the prevalence of AMR among major pathogens in clinical settings^29^ to provide the supporting data required to ensure that countries can design cost-effective, evidence-based AMR response strategies. During the first 4 years, 91 countries or territories enrolled in GLASS and data for over two million patients from 66 countries are included.^30^ Pathogens currently included in GLASS-AMR are: *Acinetobacter* spp., *Escherichia coli*, *Klebsiella pneumoniae*, *Neisseria gonorrhoeae*, *Salmonella* spp., *Shigella* spp., *Staphylococcus aureus*, and *S. pneumoniae* and a new and important component is the inclusion of antimicrobial consumption (AMC) surveillance at the national level.^31^ GLASS data is analysed based on CLSI and EUCAST breakpoints. However, Vietnam does not currently participate in this initiative.

### National surveillance studies

**Figure 4.**
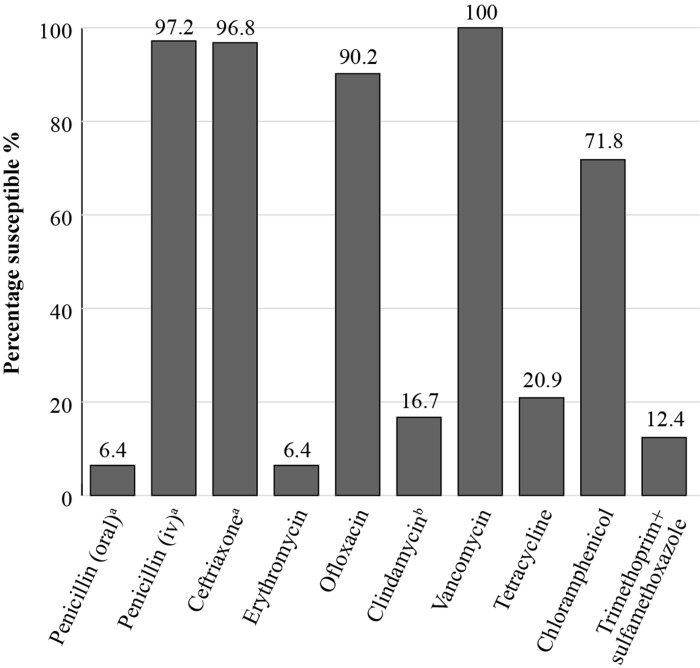
Percentage susceptibility rates based on CLSI breakpoints for antibiotics against *S. pneumoniae* isolates (*n  = *234) collected in Vietnam in 2014–15 as part of the ViPP study. ^a^218 isolates tested. ^b^233 isolates tested.

The Vietnam Pneumococcal project (ViPP)^32^ was set up to generate information which could be used to inform a new schedule for infant immunization in developing countries and support the efficient use of pneumococcal conjugate vaccines. This trial involved 1200 infants from two areas in Ho Chi Minh City, Vietnam and involved taking nasopharyngeal swabs for analysis of pneumococcal carriage and susceptibility testing of the isolates (Figure 4). A recent publication reporting the results concluded that a very high proportion of pneumococci carried in the community in Vietnam are multiresistant and that the susceptibility results from the study were comparable to those identified by SOAR.^33^

## Disease Management Guidelines

For management of the common RTIs, CAP, AOM and ABRS in Vietnam there are several country-specific local antibiotic prescribing guidelines which may not always be adhered to by clinicians. In addition, there are a range of international guidelines referred to, but these may have been created based on pathogen resistance patterns that might be very different to those in Vietnam. Examples of some of the published management guidelines in use in Vietnam are included in Table 1.

**Table 1.** Examples of local and international antibiotic prescribing guidelines referred to by physicians in Vietnam for the management of community-acquired respiratory tract infections

Local antibiotic prescribing guidelines
National treatment guideline ENT—MOH 2015^34^
National treatment guidelines for CAP—MOH 2020^35^
National treatment guidelines for AECOPD treatment 2018^37^
National treatment guidelines for implementing antimicrobial stewardship (AMS) in hospital—MOH 2020^36^
International antibiotic prescribing guidelines
IDSA 2007: Infectious Diseases Society of America. Guidelines on the management of CAP in adults^38^
BTS 2009: British Thoracic Society guidelines for the management of CAP in adults^39^
IDSA 2011 (endorsed by AAP): The management of CAP in infants and children older than 3 months of age: Clinical Practice Guidelines by the Pediatric Infectious Diseases Society and the Infectious Diseases Society of America^40^
BTS 2011: British Thoracic Society guidelines for the management of CAP in children: update 2011^41^
IDSA 2012: IDSA Clinical Practice Guideline for ABRS in children and adults^42^
AAP 2013: American Academy of Pediatrics. The diagnosis and management of AOM^43^
NICE 2014: Diagnosis and management of community- and hospital-acquired pneumonia in adults^44^
INESSS 2016: Insitut national d’excellence en santé et en services sociaux. Acute rhinosinusitis in adults^45^
INESSS 2016: Insitut national d’excellence en santé et en services sociaux Acute rhinosinusitis in children^46^
INESSS 2016: Insitut national d’excellence en santé et en services sociaux AOM in children 3 months of age or older^47^
NICE 2017: Sinusitis (acute) antimicrobial prescribing^48^
INESSS 2017: Insitut national d’excellence en santé et en services sociaux CAP in adults^49^
IDSA 2019: Diagnosis and treatment of adults with CAP. An Official Clinical Practice Guideline of the American Thoracic Society and Infectious Diseases Society of America^50^
NICE 2019: Pneumonia (community-acquired): antimicrobial prescribing^51^
WHO 2019: World Health Organization Model List of Essential Medicines^52^
EPOS 2020: EPOS 2020—European Position Paper on Rhinosinusitis and Nasal polyps^53^

Most guidelines suggest a first-line antibiotic or antibiotics along with alternative(s) and then a second-line antibiotic or antibiotics, also with an alternative(s). The first-line antibiotic is the recommended first choice that should be prescribed by the clinician following diagnosis of the infection, supported by the criteria defined by the organization or committee; alternative(s) may be provided for use in particular circumstances. For example, if the first-line antibiotic is a β-lactam then alternative suggestions may be for use in the case of allergy. The second-line antibiotic is for use if the first-line one does not achieve the anticipated outcome, and again alternative(s) may be included for use under specific circumstances.

### International antibiotic prescribing guidelines

For the management of CAP in adults and paediatrics, international guidelines referred to by clinicians in Vietnam include those from the British Thoracic Society (BTS)^41^ and the Infectious Diseases Society of America (IDSA)^38,40,50^

Both UK’s NICE guidance and Vietnam’s ‘Guidance to using antibiotic in children’ recommend amoxicillin/clavulanic acid (suspension) as alternative empirical therapy or as first-choice antibiotic in children with CAP. A first-line recommendation by the IDSA 2019 for treating adults with comorbidities and CAP is amoxicillin/clavulanic acid 875 mg/125 mg twice daily or 500 mg/125 mg given three times daily (with either a macrolide or doxycycline)^50^ however, one of the differences between the 2019 and 2007 American Thoracic Society/Infectious Diseases Society of America Community-acquired Pneumonia Guidelines is that in the 2019 guideline, macrolide monotherapy is not strongly recommended for outpatients, but rather conditionally depending on local pneumococcal resistance levels, and for standard empirical therapy for severe CAP there is stronger evidence in favour of β-lactam/macrolide combinations than β-lactam/fluoroquinolone combinations.^50^ Similar guidance is also given by Insitut national d’excellence en santé et en services sociaux (INESSS) 2017 CAP in adults.^49^

For the management of AOM, the international guidelines referred to in Vietnam include those from the American Academy of Pediatrics (AAP)^43^ and INESSS 2016: AOM in children 3 months of age or older.^47^ For the management of ABRS in adults and paediatrics the international guidelines referred to in Vietnam include those from the IDSA^42^ and INESSS 2016: Acute Rhinosinusitis in Adults,^43^ INESSS 2016: Acute Rhinosinusitis in Children^46^ and those from NICE 2017.^48^

### National antibiotic prescribing guidelines

A range of national guidelines are referred to by physicians in Vietnam, examples of which are listed in Table 1.

## Antibiotic availability

Access to antibiotics may be an issue for patients in low- and middle-income countries (LMICs) due to cost and insufficient government expenditure or support in this area. Drug supply chains may also contribute to the problem. Limited access to the most appropriate antibiotic to treat a specific infection may result in raised mortality from treatable bacterial infections, and the use of suboptimal amounts of antibiotic facilitates resistance development and allows resistant strains to persist.^54,55^ Substandard poor-quality or falsified antibiotics promote AMR and the spread of drug-resistant infections.^56^ Since poor-quality antibiotics are unlikely to contain the full dose needed to eliminate all of the infecting pathogens this would encourage resistance to develop and allow resistant strains to survive and be transmitted.^57^

In Vietnam, some currently available formulations of amoxicillin or amoxicillin/clavulanic acid, cephalosporins, macrolides, and fluoroquinolones are mentioned as first- or second-line recommendations by the RTI management guidelines and endorsed by both national and international guidelines for the treatment of a wide range of infections. Using amoxicillin/clavulanic acid as an example, this antibiotic is indicated for upper and lower RTIs, genito-urinary tract infections, skin and soft tissue infections in adults and children.^58^ Empirical treatment with antibiotics which have broad-spectrum coverage of the bacterial causes of CAP, such as amoxicillin/clavulanic acid, is used for outpatient treatment.^58^ Amoxicillin/clavulanic acid treatment recommendations for CAP include 500 mg/125 mg three times daily,^35,59^ amoxicillin/clavulanic acid 875 mg/125 mg twice daily in mild/moderate infections or a dosage of 1000 mg/125 mg given twice daily.^59^ In severe infections (including those of the lower respiratory tract) 1000 mg/125 mg is recommended three times daily in adults.^35,59^ Amoxicillin/clavulanic acid given three times daily is currently unavailable in Vietnam but is undergoing regulatory approval.

The quality of medicines, specifically antibiotics, is an important consideration for countries worldwide. The WHO launched a Global Surveillance and Monitoring System (GSMS) for substandard and falsified products.^57^ The GSMS aims to work with WHO member states to improve the quality of reporting of substandard and falsified medical products, and, importantly, to ensure the data collected are analysed and used to influence policy, procedure, and processes to protect public health, at the national, regional and the global level. Use of substandard or falsified antibiotics not only compromises clinical outcome but also risks increased AMR. The most recent summary (2013–17) reported substandard and falsified medicines in 46 member states (including Vietnam) and antibiotics represent 16.9% of all products reported, second only to malaria drugs (19.6%).^57^

## Local insight

### Clinician expert comments

Vietnam is a hotspot of AMR in Asia, where the burden of resistant infections is disproportionate. In particular, the susceptibilities of the two most common respiratory pathogens, responsible for many of the incidences of CAP in both adults and children, *S. pneumoniae* and *H. influenzae*, are decreasing. For *S. pneumoniae*, increasing resistance is seen to all antibiotics, including penicillins, cephalosporins, fluoroquinolones and macrolides. In efforts to address the problem of rising AMR worldwide, one of the strategic objectives outlined by the WHO was to strengthen the knowledge and evidence base through surveillance. Early studies aimed to implement AMR surveillance with national and international efforts in Vietnam. These showed the importance of local susceptibility data, providing solid evidence on the high AMR pattern in Vietnam. In the most recent study (Viet Nam Resistance: VINARES 2016–17), which was a hospital antibiotic resistance surveillance study covering a network of hospitals, results showed that 58% (663/1136) of *S. pneumoniae* had reduced susceptibility to penicillin and 88% (804/911) of *H. influenzae* were resistant to ampicillin;^60^ local surveillance on CA-RTI also revealed similar alarming messages. In the SOAR studies, as described in this paper, Vietnam had the lowest susceptibilities of *S. pneumoniae* and *H. influenzae* to most of the antibiotics tested among the four Asian countries reported. It is noticeable that only 1.2% of *S. pneumoniae* isolates were susceptible to intravenous penicillin, 59.6% were susceptible to amoxicillin whereas only 11.2% of *H. influenzae* isolates showed susceptibility to ampicillin.^18^ These pathogens are commonly involved in other community-acquired infections such as AOM and ABRS, but minimal data are available so far for these infections specifically. Therefore, it is even more challenging to have optimal antibiotic management in Vietnam when there is limited information available regarding the AMR profiles.

The Vietnamese Ministry of Health has issued a general antibiotic treatment guideline and also specific guidelines for CA-RTI, AOM, and ABRS for both adults and children. However, guidelines on infectious diseases at the national level have had limited impact, with low rates of adherence. In a multicentre study on CAP, widespread use of intravenous third-generation cephalosporins was observed (29.3% as monotherapy and 40.4% as combination therapy) regardless of infection severity.^61^ Irrational antibiotic use along with guideline non-compliance might be explained by the lack of local microbiology data-driven guidance, patient-expectation oriented prescribing behaviour, lack of consultation time with each patient and promotion of broad-spectrum antibiotic use by pharmaceutical companies.^62^ Therefore, antimicrobial stewardship teams in Vietnam should elaborate the guidelines with local microbiological data, as well as integrate and disseminate the guidelines to doctors.^36^

Vietnam is an emerging pharmaceutical market with antimicrobial consumption ranked 11th out of 76 countries.^13^ Stratifying antibiotics by the WHO Access framework, Vietnam has a high rate of consumption of Watch-group antibiotics, particularly in children. A 2018 study in pharmacies showed that 54.8% of pharmacy encounters, 53.0% of days of treatment and 53.6% of DDD in children were Watch-group antibiotics. Vietnam had the largest increase in Watch antibiotic consumption (10.6 DDDs per 1000 inhabitants per day) our of 75 countries from 2000 to 2015.^13^

The Vietnamese Pharmaceutical Law (2005) made antibiotics prescription-only drugs. Despite this, antibiotic dispensing without prescription is still a major problem in Vietnam. A common reason for buying antibiotics is mild acute respiratory infection (ARI) without fever.^63^ A study involving children <5 years of age in rural Vietnam showed that although most caregivers knew that antibiotics were not required for mild ARI without fever, such as the common cold, 71.0% of children were actually prescribed them and most (82%) of the unnecessary antibiotics had been recommended by a healthcare professional.^63^ Inappropriate antibiotic dispensing was considered the main reason for the increase in the AMR rate of *S. pneumoniae* in the community. A trend towards prescribing of broader spectrum antibiotics was common, with cephalosporins gradually replacing penicillins from 1991 to 2007.^12^

Pharmacies in Vietnam are rarely penalized for antibiotic dispensing without prescription. The accessibility to antibiotics is only controlled by pre-authorization policy as recommended in the national antimicrobial stewardship guideline. More-restrictive measures are needed to control, monitor, and optimize antibiotic use, both in the community and in hospital.^64,65^ Surveillance data are therefore critical to inform infection treatment guidelines, monitor trends, and to assess interventions, although most surveillance programmes are passive and pathogen-based, which will inevitably lead to bias. Addition of clinical and patient outcome data would provide considerable added value to pathogen-based surveillance, as would continuously monitoring local antibiotic use and bacterial resistance. In addition, in terms of guidelines, clinicians need to move from expert-based guidelines to evidence-based ones and institutions also need to follow strict infection control.

## Conclusions

In an era of rising AMR throughout the world, this paper aims to define areas where action is required to address AMR by analysing and understanding the current situation within a country or region. Information is presented for Vietnam concerning antibiotic use and prescribing, approach to AMR, availability of local susceptibility data, use of international and/or local management guidelines and how these link to antibiotic availability. To our knowledge this is the first time this information has been reviewed and presented in detail by country.

Antibiotic use in Vietnam is extremely high and continues to rise. OTC sale of antibiotics plays an important role in increasing the rate of AMR. Education and enforcement concerning restricting OTC use of antibiotics will be needed for pharmacists and the same topics along with the harm of overuse and misuse of antibiotics would form the basis of a country-wide education campaign for the public, which would need to be repeated to reinforce these messages.

Antibiotic susceptibility is low for most antibiotic classes in Vietnam; the fluoroquinolone antibiotics have so far retained higher levels of activity, although guidelines and regulatory bodies urge caution, restricting their use to limited situations due to serious safety concerns.

There are some international and local surveillance studies for CA-RTI pathogens in Vietnam but development of a coordinated national antibiotic surveillance system for Vietnam in which the data are collected by accredited laboratories under international standards, followed by data sharing would provide a centralized accurate picture of antibiotic susceptibility throughout the country. In addition, according to the expert clinician comments, whilst surveillance studies include clinical isolates from CAP patients, these pathogens are commonly involved in other community-acquired infections such as AOM and ABRS, but minimal data are available so far for these specific infections.

Whilst a range of international and Vietnamese guidelines is utilized by clinicians in Vietnam, a more standardized inclusive approach could be helpful to develop local country-specific guidelines avoiding any reliance on the international guidelines which have been developed in countries or regions with markedly different susceptibility patterns to those that are very specific to Vietnam. These guidelines would be based on the up-to-date surveillance data of isolates from community-acquired infections which would make them more locally relevant for clinicians, reiterating the Consensus Principles as described in the introductory paper to this Supplement.^2^ This would pave the way for improved adherence and a higher level of appropriate antibiotic prescribing in CA-RTIs which could, in turn, potentially limit AMR development and improve clinical outcomes for patients.
